# Correlation of catecholamine content and clinical influencing factors in depression among psoriasis patients: a case-control study

**DOI:** 10.1186/s13030-022-00245-2

**Published:** 2022-08-10

**Authors:** Si-Qi Long, Jing Fang, Hui-Ling Shu, Deng-Mei Xia, Zheng-Qun Wang, Wen-Yao Mi, Xue-Li Zhang, Chang-Qiang Li

**Affiliations:** 1 Department of Dermatology, Affiliated Hospital of Southwest Medical University, No. 25 Taiping Road, LuZhou, 646000 China; 2 Department of Dermatology, Qingbaijiang District People’s Hospital of Chengdu, No.9 Fenghuang East Fourth Road, Chengdu, 610300 China; 3 Department of Dermatology, People’s Hospital of Chongzhou, No.318 Yongkang East Road, Chongzhou, 611230 China; 4 Department of psychiatry, Affiliated Hospital of Southwest Medical University, No.25 Taiping Road, LuZhou, 646000 China

**Keywords:** Case control study, Psoriasis vulgaris, Depression, Catecholamine

## Abstract

**Objective:**

Our study sought to investigate the clinical influencing factors of psoriasis patients with depression, and analyze whether the content of monoamine neurotransmitters in plasma was correlated with depression incidence among psoriasis patients.

**Methods:**

Ninety patients with psoriasis and 40 healthy volunteers (aged from18 to 60) were recruited and interviewed with a piloted questionnaire in both groups to obtain relevant information. The catecholamine in plasma from the two groups was analyzed by radioimmunoassay. The data were analyzed by SPSS statistical software.

**Results:**

The mean Hamilton Depression Scale (HAMD) and mean Athens Insomnia Scale (AIS) scores of the psoriasis patients were higher than the control group. Dopamine content in the plasma was lower (comparing psoriasis patients without depression and the control group, and was negatively correlated with HAMD, AIS, and Psoriasis Area and Severity Index (PASI) scores in the psoriasis patients with depression. There was no significant difference in the epinephrine and norepinephrine contents in all groups. PASI scores were positively correlated with HAMD scores in psoriasis patients. The low dopamine content, Dermatology Life Quality Index, and high PASI scores were the risk factors for depression among the psoriasis patients.

**Conclusion:**

Psoriasis patients have a significantly higher risk of depression than healthy people, and higher PASI scores were linked to a higher incidence of depression. The dopamine levels of patients were influenced by both psoriasis and depression. The risk factors for depression in psoriasis patients are low dopamine levels in the plasma, severe skin lesions, and lower quality of life.

## Introduction

Psoriasis is a chronic inflammatory skin disease mainly manifested by erythema scales, affecting approximately 1–8% of the global population [1, 2]. Two peak ages of onset are considered for the disease; the early age of onset is between 16-22 years, and the other is 57-60 years [3]. The disease is often divided into four different types: the vulgaris, the erythroderma, the pustular subtype, and the arthropathy, according to the clinical characteristics. Generally, self-confidence of psoriasis patients is affected by the physical appearance of skin lesions, such as plaque and scales, although psoriasis generally does not have a great impact on the physical health of patients. Moreover, low self-esteem, anxiety, and depression seriously affect the quality of life of patients [4]. The mechanism of psoriasis vulgaris, the most common type of psoriasis, is jointly involved in the nervous-endocrine-immune systems [5], and its onset is often related to infection [6], heredity [7], endocrine [8], spiritual [9], and environmental [10] factors, among which neuroimmune regulation is considered as an important link [5]. Some studies have shown that neurotransmitters in the nerve fibers of skin and various skin cells include 5-HT, NE, DA [11]. Catecholamines, as monoamine neurotransmitters, are mainly involved in mental activities and emotional control in humans, and have been proven to be closely related to the onset of depression [12]. However, catecholamine content in psoriasis patients with depression remain unknown to date, and the patients’ quality of life and economic status have not been evaluated in previous reports. However, the change of the catecholamine content in serum could be used as a reference index for diagnosing depression in psoriasis patients as peripheral blood may reflect that in the central nervous system. Therefore, we investigated the clinical influencing factors of patients with psoriasis vulgaris complicated with depression, and analyzed whether the content of monoamine neurotransmitters in plasma is correlated with depression among psoriasis patients. Moreover, providing new ideas and methods may improve early recognition, diagnosis, and treatment of psoriasis complicated with depression.

## Material and methods

### Patients

Ninety psoriasis patients aged 18-60 who were referred to the Department of Dermatology at the Affiliated Hospital of Southwest Medical University were included in the study. The study protocol was approved by the ethics committee of the affiliated Hospital of Southwest Medical University. Written informed consent was obtained from all participants, and the control group included 40 healthy volunteers aged 18-60 referred to the physical examination center of the hospital. The inclusion criteria were conformed diagnostic criteria of psoriasis vulgaris; age 18-60 years; BMI between 18 and 25; be able to understand all the contents of the questionnaire and the informed; complete the questionnaire; and participate in the study voluntarily. Exclusion criteria were having other skin conditions; being treated with hormone medications; patients with intracranial lesions and severe heart, liver, renal insufficiencies and other major diseases; pregnant, breastfeeding, and menstrual women; and patients with high blood pressure, diabetes, and other metabolic diseases.

### Methods

Questionnaire survey was conducted in both groups to obtain basic patient information, Hamilton Depression Scale (HAMD, 24 HAMD score ≥18 was defined as accompanied by depressive symptoms), and Athens Insomnia Scale (AIS, score ≥7 was classified as insomnia). In the psoriasis vulgaris group, the contents of the questionnaire also included the course of the disease, Psoriasis Area and Severity Index (PASI, mild 0-10, moderate 11-20, severe >20), and Dermatology Life Quality Index (DLQI, the higher the score, the worse the patient's quality of life). All participants in the study fasted for 8 hours and had venous blood taken at 8 a.m. However, the psoriasis patients had not received anti-psoriasis treatment for one month prior to blood extraction, and the plasma catecholamine content was analyzed by radioimmunoassay.

### Statistics

In this study, all analyses were performed by SPSS software. Continuity variables were described using median and interquartile spacing (Medians, IQR)，Categorical variables are described in percentages；Comparison of difference between two groups of continuous variables using Mann-Whitney Rank Sum test (Mann-Whitney U)；Chi-square test was used to compare the difference of composition ratio between two and three groups；Spearman's rank correlation test was used to test the correlation of two continuous variables, and ANOVA was used to compare the three groups of continuous data. The relationship between plasma catecholamine and psoriasis with depression and risk factors of depressive symptoms in patients with psoriasis were analyzed by logistic regression. The significance test was =0.05.

## Results

### Comparison of clinical data between groups

According to the PASI scores, in the 90 patients with psoriasis vulgaris, 77% were severe, and 23% were moderate. According to the clinical symptoms, HAMD scores, and PASI scores of the patients, depression symptoms accounted for 20% of patients with psoriasis vulgaris, including 2 patients with moderate psoriasis vulgaris and depression, moderate psoriasis vulgaris without depression in 20 patients; 16 patients with severe psoriasis vulgaris and depression. Severe psoriasis vulgaris without depression was reported in 52 patients.

In this study, there was no evidence of differences in sex (*P*=0.271), family conditions (*P*=0.124), average age (*P*=0.709), and the content of norepinephrine in the plasma (*P*=0.159) between the case and control groups. The mean HAMD scores (*P*=0.034) and mean AIS scores (*P*=0.001) of the case group were significantly higher than that of the control group, while the mean content of dopamine (*P*=0.004) and adrenaline (*P*=0.007) in the plasma were lower than that of the control group. (Table 1)

**Table 1:** Comparison of basic data between the case group and the control group

	The Psoriasis group	The Control group	*z*或*χ*^*2*^	*P*
Number, n	90	40		
Age (year)	44(25-53)	44(31-50)	-0.374	0.709
Sex (male), n(%)	67(74)	26(65)	1.213	0.271
Course of disease (month)	78(12-120)			
Poor economic condition, n (%)	21(23)	4(10)	2.369	0.124
HAMD score	3(0-9)	2(0-4)	-2.115	0.034
PASI score	31(21-39)			
DLQI score	5(2-9)			
AIS score	2(0-9)	0(0-1)	-3.407	0.001
Phenylephrine (pg/ml)	30.84(29.16-33.29)	36.77(29.79-41.18)	-2.679	0.007
Norepinephrine (pg/ml)	149.28(141.78-161.13)	154.52(142.51-163.88)	-1.407	0.159
Dopamine (pg/ml)	53.73.(49.73-56.67)	57.08(52.78-63.11)	-2.916	0.004

*HAMD* Hamilton Depression Scale*, PASI* Psoriasis Area and Severity Index*, DLQI* Dermatology Life Quality Index*, AIS* Athens Insomnia Scale

### Comparison of the catecholamine content and other clinical variables in three groups (psoriasis with depression group, psoriasis without depression group, and the control group)

The content of dopamine in the plasma of the psoriasis with depression group was lower than that of the psoriasis without depression group and the control group (*P*=0.001, Table 2). However, the average dopamine content in the plasma of the psoriasis patients without depression was lower than that of the control group, although the difference was not statistically significant (z=-1.931, P>0.05). There were no significant differences in sex (*P*=0.531), economic conditions (*P*=0.097), the mean content of adrenaline (*P*=0.308), and norepinephrine (*P*=0.497) in the plasma among the three groups. However, there was evidence that age (*P*=0.003) plays a role in the three groups. As for psoriasis patients, the AIS scores (*P*=0.0001), DLQI scores (*P*=0.0001), PASI scores (*P*=0.011), and course of disease (*P*=0.026) in those with depression were significantly higher than those without depression, and family members of the patients with depression were less likely to have the same disease (*P*=0.097).

**Table 2:** Comparison of clinical data among the three groups

	Psoriasis with depression	Psoriasis without depression	The control group	*Z、F或*χ^2^	*P*
Number，n	18	72	40		
Dopamine (pg/ml)	50.15(47.01-51.43)	54.74(51.54-57.93)	57.08(52.78-63.11)	7.359	0.001
Phenylephrine (pg/ml)	29.84 (29.12-31.53)	31.78(29.17-34.30)	36.77(29.79-41.18)	1.190	0.308
Norepinephrine (pg/ml)	149.22(140.05-158.83)	149.28(142.99-161.13)	154.52(142.51-163.88)	0.704	0.497
Age (year)	48(44-67)	41(24-52)	44(31-50)	6.002	0.003
Sex (male),n(%)	13(72)	54(75)	26(65)	1.268	0.531
Poor economic condition, n(%)	6(33)	15(21)	4(10)	4.672	0.097
Course of disease (month)	120(45-240)	72(6-120)		-2.226	0.026
PASI score	39(31-49)	29(18-37)		-2.542	0.011
DLQI score	11(10-17)	4(2-7)		-6.152	0.000
Family history, n(%)	6(33%)	9(13)		4.500	0.034
The number of insomnia, n(%)	11(61)	12(17)	3(7.5)	23.420	0.000

*PASI* Psoriasis Area and Severity Index*, DLQI* Dermatology Life Quality Index

### Comparison of catecholamine content in the plasma of psoriasis patients with and without depression according to PASI scores

In patients with moderate psoriasis regardless of depression incidence, there was no difference in the contents of dopamine (*P*=0.209) and adrenaline (*P*=0.253) in the plasma. However, the dopamine content in the plasma of the patients with depression (*P*=0.001) was significantly lower than those without depression, although there was no difference in the content of adrenaline (*P*=0.386) and norepinephrine (*P*=0.603) in the plasma in patients with severe psoriasis (Tables 3 and 4).

**Table 3:** Comparison of catecholamine content between moderate psoriasis patients with and without depression

	With depression	Without depression	*Z*	*P*
Dopamine (pg/ml)	48.1(45.15-50.32)	55.24(49.25-58.07)	-1.257	0.209
Phenylephrine (pg/ml)	29.97(29.37-30.57)	32.52(29.40-40.14)	-1.143	0.253
Norepinephrine (pg/ml)	161.52(146.60-176.430)	149.06(141.055-160.748)	-1.028	0.304

**Table 4:** Comparison of catecholamine content between severe psoriasis patients with and without depression

	With depression	Without depression	*Z*	*P*
Dopamine (pg/ml)	50.16(47.23-52.32)	54.74(51.86-57.26)	-3.326	0.001
Phenylephrine (pg/ml)	29.83(29.04-31.84)	30.84(29.01-33.24)	-0.868	0.386
Norepinephrine (pg/ml)	149.220(139.715-155.755)	150.01(143.035-161.242	-0.521	0.603

### Correlation analysis of the catecholamine (dopamine) content in the plasma and other clinical variables in psoriasis patients with depression

**Fig 1:**
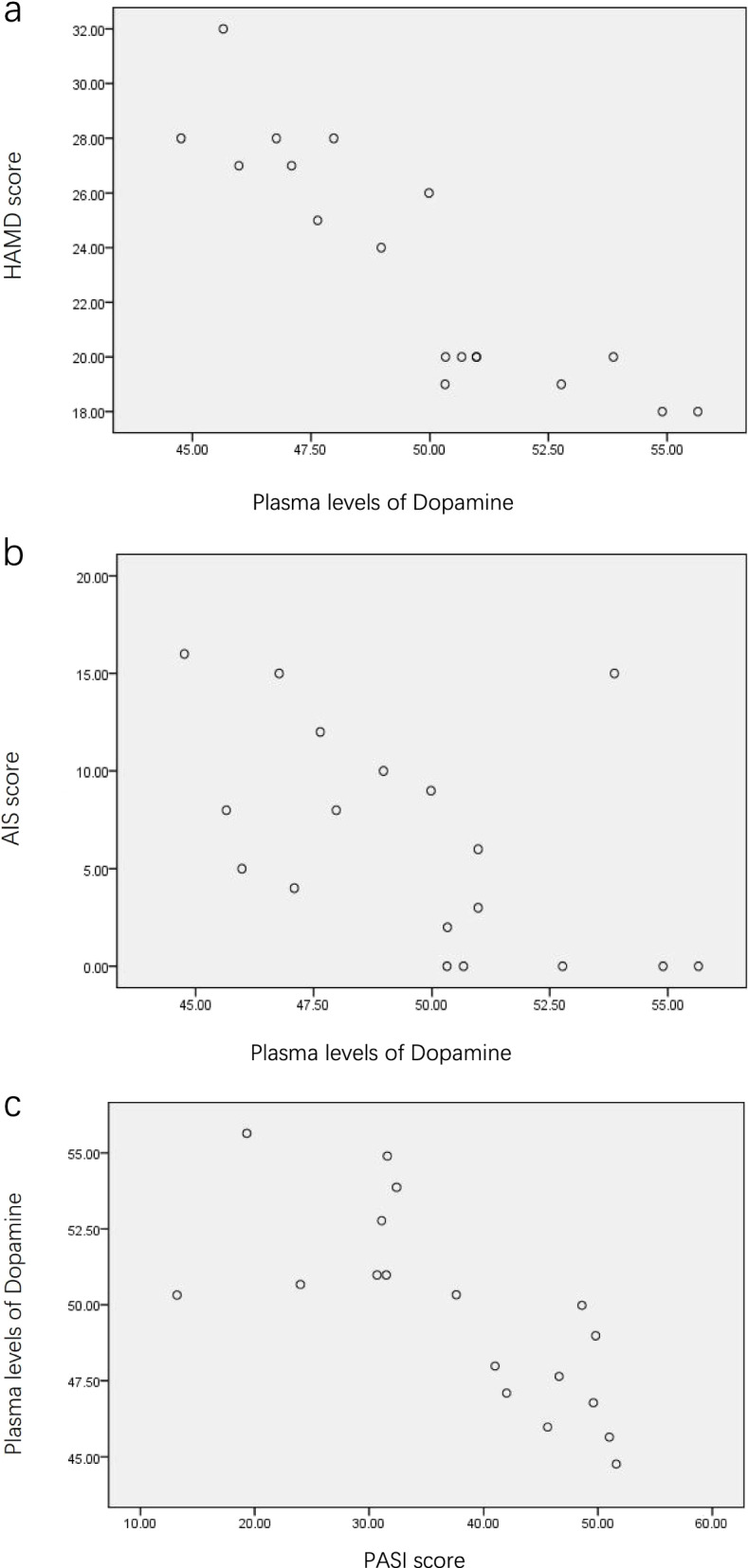
**a** Plasma levels of Dopamine are negatively correlated with HAMD score. *HAMD* Hamilton Depression Scale. **b** Plasma levels of Dopamine are negatively correlated with AIS score. *AIS* Athens Insomnia Scale. **c** Plasma levels of Dopamine are negatively correlated with PASI score. *PASI* Psoriasis Area and Severity Index

Dopamine content in the plasma was negatively correlated with HAMD scores (r=-0.882, P<0.05), AIS scores (r=-0.514, P<0.05), and PASI scores (r=-0.731, P<0.05), while it was not significantly correlated with the course of disease, age, economic conditions, and quality of life of the patients (Fig. 1a, b, c and Table 5).

**Table 5:** The relationship between plasma dopamine level and course of disease, age, quality of life, and economic condition of patients

	Dopamine (pg/ml)	
	*r*	*P*
Age (year)	-0.443	0.066
Course of disease (month)	-0.022	0.930
Poor economic condition, n(%)	-0.296	0.233
DLQI score	-0.233	0.352

*DLQI* Dermatology Life Quality Index

### Correlation analysis of the dopamine content and other clinical variables in psoriasis patients without depression

There was no significant correlation between dopamine content and HAMD scores, AIS scores, PASI scores, DLQI scores, age, course of disease, and economic condition in the patients (Table 6).

**Table 6:** The relationship between dopamine content and HAMD score, AIS score, disease, age, quality of life, and economic condition of patients

	Dopamine (pg/ml)	
	*r*	*P*
HAMD score	-0.056	0.641
AIS score	0.041	0.735
PASI score	0.171	0.152
DLQI score	-0.008	0.949
Age (year)	0.120	0.317
Course of disease (month)	-0.057	0.636
Poor economic condition, n(%)	0.054	0.654

*HAMD* Hamilton Depression Scale*, AIS* Athens Insomnia Scale, *PASI* Psoriasis Area and Severity Index*, DLQI* Dermatology Life Quality Index

### Correlation analysis of PASI, HAMD, DLQI, and AIS scores in patients with psoriasis vulgaris

**Fig. 2:**
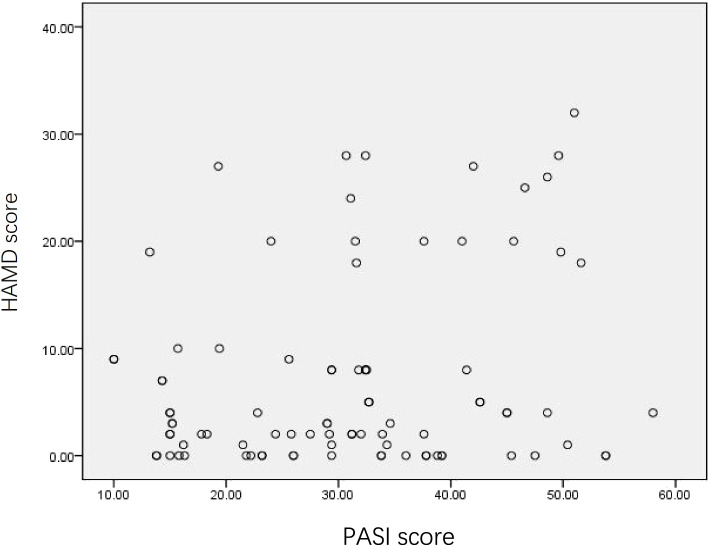
PASI score is positively correlated with HAMD score. *PASI* Psoriasis Area and Severity Index. *HAMD* Hamilton Depression Scale

PASI score was positively correlated with HAMD score (*r*=-0.236, *P*=0.025) (Fig. 2), but not significantly correlated with DLQI score and AIS score (Table 7) in the patients.

**Table 7:** The relationship between PASI score and course of HAMD and AIS scores of patients

	PASI score	
	*r*	*P*
AIS score	-0.108	0.312
DLQI score	0.152	0.154

*AIS* Athens Insomnia Scale, *DLQI* Dermatology Life Quality Index

### Univariate analysis and logistic regression multivariate analysis of the psoriasis patients with depression

There were statistically significant differences in age, mean course of disease, PASI score, family history, DLQI score, and AIS score between the psoriasis patients with depression and those without depression (Table 8). However, there was no significant differences in sex and economic conditions. The analysis showed that the low dopamine content was a risk factor for depression among patients with psoriasis (OR 1.448, 95%CI: 1.033-2.029; *P* < 0.05). Other risk factors included high PASI scores (OR 0.904, 95%CI: 0.845-0.968); (*P*<0.05), lower quality of life, DLQI scores (OR 0.601, 95%CI: 0.429-0.842; *P* < 0.05). There was no significant correlation with sex, age, course of disease, family history of psoriasis, sleep, economic conditions, and the content of adrenaline and norepinephrine.

**Table 8:** Univariate and multivariate analyses of the two groups

	The Psoriasis group	The Control group	*P*	OR(95%CI)	*P’*
Number, n	18	*72*			
Age (years)	48(44-67)	41(24-52)	0.005	0.954(0.895-1.017)	0.151
Sex, n(%)	13(72)	54 (75)	0.809	3.405(0.207-56.081)	0.391
Poor economic condition, n(%)	6(33%)	15(21)	0.262	0.344(0.021-5.701)	0.456
Family history, n(%)	6(33%)	9(13)	0.034	0.007(0.000-4.174)	0.128
Course of disease (month)	120(45-240)	72(6-120)	0.026	0.996(0.984-1.009)	0.556
AIS score	6(0-11)	1(0-9)	0.048	0.995(0.729-1.358)	0.977
PASI score	39(31-49)	29(18-37)	0.011	0.904(0.845-0.968)	0.004
DLQI score	11(10-17)	4(2-7)	0.000	0.601(0.429-0.842)	0.003
Dopamine (pg/ml)	50.15(47.01-51.43)	54.74(51.54-57.93)	0.000	1.448(1.033-2.029)	0.032
Norepinephrine (pg/ml)	149.22(140.05-158.83)	149.28(142.99-161.13)	0.928	1.002(0.913-1.101)	0.961
Epinephrine (pg/ml)	29.84(29.12-31.53)	31.78(29.17-34.30)	0.155	0.952(0.787-1.151)	0.611

*AIS* Athens Insomnia Scale, *PASI* Psoriasis Area and Severity Index*, DLQI* Dermatology Life Quality Index

## Discussion

Psoriasis is a chronic inflammatory skin disease, manifesting mainly erythemato-squamous plaques [2], and its occurrence and development are closely related to the expression imbalance of genes related to immune dysfunction [13], vascular injury [14] and abnormal signal transduction pathway [15]. Biological and seasonal factors are the two most common causes, among which winter and spring play an important role.

In our study, the sleep quality of patients with psoriasis was significantly affected, and their quality of life was lower than that of the control group. Skin lesions of patients with psoriasis may often be accompanied with itching and irritability, and the patients were able to be self-abased, when the skin lesions occur in the exposed parts. Furthermore, sexual activities and drive of such patients tend to be affected if psoriasis occurs in the perineum and other private parts. Some other studies have shown that psoriasis may lead to male erectile dysfunction, and may even be an independent risk factor for erectile dysfunction [16]. Therefore, patients are prone to insomnia and decreased quality of life compared with the control group as pruritus of the skin lesions manifested more at night. Thus, patients are affected by worry, depression, anxiety, and other negative emotions. The course of psoriasis is fairly long and recurrent, and although there are several various treatment methods, curability and efficacy are elusive, thereby significantly affecting health and confidence. Because of the long-term trouble caused by the disease, the patients often showed symptoms of depression and anxiety, which affected their general quality of life. Recently, with the development of society and the continuous improvement of living standards, the biopsychosocial medical model has attracted increasing attention, and psoriasis patients’ mental health had also been given focus.

Female patients with psoriasis are more likely to suffer from depression [17, 18], which is consistent with Tian Z et al. [19] 's on Chinese people, suggesting that there was no significant difference in depression among patients with psoriasis on different sexes. Comparing national conditions at home and abroad could be found that the number of patients with psoriasis is almost equal between men and women beyond the seas [20]. However, in some areas, such as the south bank of the Atlantic, Pacific, and New England region, the prevalence rate of psoriasis in women was even higher than that in men. While in China, however, the opposite was true [21]. In terms of the sample size, sex composition ratio was different at home and abroad, and the traditional concept thought that women pay more attention on their external image than men do. However, with the improvement of China's living standards, men's attention to external image is increasing.

The study found that psoriasis patients with a long course were more prone to depression than those with a short course, which was the same with the result of Lakshmy et al [22]. Moreover, it has also been reported that psoriasis patients with short course are more prone to depression than those with long course, such as the study of Tian Z et al [19]. Repeated attacks of the disease often turn initial hopes of a cure into disappointment. Repeated visits to the doctor often lead to anxiety and, over time, depression. Therefore, the knowledge popularization and health education of the newly diagnosed patients are particularly important in clinical practice. The body management concept of "with chronic diseases and self-discipline as medicine" should be instilled into every patient with psoriasis.

Tian Z et al. [19] believed that the onset of depression among psoriasis vulgaris was not related to age, but was related to the age at which psoriasis occurred. Conversely, the result of the study showed that older patients had a higher prevalence of depression. In terms of contemporary medical conditions and prospects, young patients may be more willing to believe that the medical community can overcome the "psoriasis" problem in the near future; In contrast, the older patients, based on rich social experience and lack of new knowledge, were more worried and pessimistic than the younger ones.

Studies have confirmed that dopamine, norepinephrine, and other monoamine neurotransmitters have a wide range of biological activities, which can participate in physiological responses in mental activities, mood, sleep and other central system. The biological basis for depression may decrease or lower the function of these neurotransmitters in the synaptic gap in the brain [23]. In 1979, it was proposed that low levels of norepinephrine in the brain could lead to depression [24], and the hypothesis was confirmed later. However, other studies have reached the opposite conclusion, which shows that the content of NE in depressed patients was higher than that of normal people (Lake, 1982). There also was an inference that tension and anxiety can activate the hypothalamic-pituitary-adrenal axis, producing more catecholamines, which could participate in the occurrence and development of psoriasis. Our patients were older patients, chronic psoriasis, and often felt depression because they realized that the disease is relapses easily and cannot be cured. The study of Violanti JM et al. has shown that chronic anxiety can lead to depression [25]. Moreover, the horny cell of the patients with psoriasis can secrete IL-6, TNF alpha, IL-1 alpha, glucocorticoids, among others. These inflammatory factors could affect the central nervous system, promoting the occurrence of the depression, while a large number of inflammatory cytokines could produce excessive inflammation, destroy neurogenesis, lead to neuronal dysfunction, and promote the development of depression [26, 27]. Additionally, during the whole chronic course of psoriasis, the secretion of neurotransmitters such as NE and DA was abnormal due to long-term negative emotional stress. Therefore, the patients with psoriasis included in our study were all in-patients with severe inflammatory response and high levels of inflammatory factors, which damaged structures such as neurons, resulting in decreased the content of catecholamine transmitters, and ultimately suffered depression.

Studies found that catecholamine neurotransmitters such as DA and NE could activate immune cells (lymphocytes, macrophages, and natural killer cells), had a regulatory effect on the activity of lymphocytes and macrophages by binding to the corresponding receptors on immune cells [28], and were important components of the nerve-endocrine-immune regulatory system, which play a significant role in the occurrence and development of psoriasis.

In the study, the level of dopamine in the blood of patients with depression was lower than that of patients without depression in the patients with severe psoriasis, and the higher the PASI score, the more likely the patients were to have depression. Further, there was no significant correlation between depression and non-depression in patients with moderate psoriasis. The role of dopamine as an intermediate transmitter in the mechanism of co-pathogenesis of psoriasis and depression, and the drugs used to alter abnormal levels of catecholamines in plasma or the central nervous system to treat both psoriasis and depression are important points to be elucidated to improve the condition of psoriasis patients complicated with mental abnormalities.

There were some evidences that the prevalence ratios of psoriasis with depression varies from 9.7% to 96% [29, 30]. In our study, psoriasis patients with depression accounted for only 20%, which was lower than most previous studies. The is because of the differences in the scales used to evaluate depression (such as PHD-9 and GAD-7 scales), and the judgment among evaluators. In the study, the alternative rating scale was selected and completed with the assistance of the physicians in charge of professional psychiatry, which would be more objective. Moreover, the study only studied psoriasis vulgaris, excluding the other three more serious types.

Some study limitations were: 1. This was a cross-sectional study, and the causal relationship between psoriasis vulgaris and depressive symptoms, the dopamine levels and other risk factors cannot be determined. 2. In order to better evaluate the skin lesions, we only investigated and evaluated psoriasis vulgaris, and the other three more severe types were not included in the study; 3. The study was a single-center study with a small sample size, especially for psoriasis patients with depression. The research should be carried out with a large sample or a multi-center study in the future.

## Conclusion

The results revealed that patients with psoriasis vulgaris have a significantly higher risk of depression than healthy people, and the higher the PASI scores, the more likely they were to have depression. The dopamine levels of patients were influenced by both psoriasis and depression. The risk factors for depression in psoriasis patients were low levels of dopamine in the plasma, severe skin lesions, and lower quality of life. Psoriasis patients with depression had a lower dopamine content, which was closely related to sleep quality, severity of skin lesions, and depression score.
